# Clinical efficacy and molecular biomarkers in a phase II study of tucidinostat plus R-CHOP in elderly patients with newly diagnosed diffuse large B-cell lymphoma

**DOI:** 10.1186/s13148-020-00948-9

**Published:** 2020-10-23

**Authors:** Mu-Chen Zhang, Ying Fang, Li Wang, Shu Cheng, Di Fu, Yang He, Yan Zhao, Chao-Fu Wang, Xu-Feng Jiang, Qi Song, Peng-Peng Xu, Wei-Li Zhao

**Affiliations:** 1 Shanghai Institute of Hematology, State Key Laboratory of Medical Genomics, National Research Center for Translational Medicine At Shanghai, Ruijin Hospital Affiliated To Shanghai Jiao Tong University School of Medicine, Shanghai, China; 2 Pôle de Recherches Sino-Français en Science du Vivant Et Génomique, Laboratory of Molecular Pathology, Shanghai, China; 3 Department of Pathology, Ruijin Hospital Affiliated To Shanghai Jiao Tong University School of Medicine, Shanghai, China; 4 Department of Nuclear Medicine, Ruijin Hospital Affiliated To Shanghai Jiao Tong University School of Medicine, Shanghai, China; 5 Department of Radiology, Ruijin Hospital Affiliated To Shanghai Jiao Tong University School of Medicine, Shanghai, China

**Keywords:** Diffuse large B-cell lymphoma, Double expressor lymphoma, Histone deacetylase inhibitor, Tucidinostat, CREBBP/EP300

## Abstract

**Background:**

Elderly patients with diffuse large B-cell lymphoma (DLBCL) present with poor clinical outcome and intolerance to intensive chemotherapy. Histone deacetylase inhibitors (HDACIs) show anti-lymphoma activities and can be applied to treat DLBCL. This study aimed to evaluate efficacy and safety of oral HDACI tucidinostat (formerly known as chidamide) plus R-CHOP (CR-CHOP) in elderly patients with newly diagnosed DLBCL (International Prognostic Index ≥ 2).

**Results:**

Among 49 patients, the complete response rate was 86%, with overall response rate achieving 94%. The 2-year progression survival (PFS) and overall survival (OS) rates were 68% (95% CI 52–79) and 83% (95% CI 68–91). Comparing with historical control (NCT01852435), the 2-year PFS and OS rates of double-expressor lymphoma phenotype (DEL) were improved, and negative prognostic effect of histone acetyltransferases *CREBBP*/*EP300* mutations was also mitigated by CR-CHOP. Grade 3–4 neutropenia was reported in 171, grade 3–4 thrombocytopenia in 27, and grade 3 anemia in 11 of 283 cycles. No grade 4 non-hematological adverse event was reported.

**Conclusion:**

CR-CHOP is effective and safe in elderly patients with newly diagnosed DLBCL. Relevance of DEL phenotype and molecular biomarkers on CR-CHOP response warrants further investigation in DLBCL.

*Trial registration* ClinicalTrial.gov, NCT02753647. Registered on April 28, 2016.

## Background

Diffuse large B-cell lymphoma (DLBCL) represents the most common subtype of non-Hodgkin’s lymphoma and is heterogeneous in clinical, immunophenotypic, and molecular features. More than 50% of DLBCL are elderly patients older than 60 years at diagnosis [1] and have advanced stage disease, intermediate- to high-risk International Prognostic Index (IPI) and adverse clinical outcome [2–4]. The progression-free survival (PFS) and overall survival (OS) rates were only 54% and 58% at 5 years upon treatment with R-CHOP (rituximab, cyclophosphamide, doxorubicin, vincristine, and prednisone) [2], remarkably inferior to those of DLBCL patients younger than 60 years [5]. Moreover, most of the elderly patients are ineligible for intensive chemotherapy and/or hematopoietic stem cell transplantation, making effective and safe treatment as an unmet need in this subset of DLBCL.

In addition to clinical parameters, several biological features have been revealed as important prognostic indicators in DLBCL. Based on cell of origin, DLBCL can be classified as germinal center B-cell like (GCB), activated B-cell like (ABC), and unclassified, the latter two referred as non-GCB phenotype [6, 7]. Patients with non-GCB have a worse prognosis than those with GCB phenotype [6]. Double-expressor lymphoma (DEL) is defined as co-expression of BCL2 (> 50%) and MYC (> 40%) proteins by immunohistochemistry without underlying rearrangements [1]. DEL patients respond poorly to standard R-CHOP or intensive chemotherapy followed by stem cell transplantation [8]. As for molecular alterations, epigenetic gene mutations are frequently observed in DLBCL, mainly including histone methyltransferases *KMT2C*, *KMT2D*, and *EZH2*, histone acetyltransferases *CREBBP*, *EP300*, and *IRF4*, chromatin remodeler *HIST1H1E* and *ARID1A*, and DNA methylation gene *TET2* [9]. GCB DLBCL with *CREBBP*, *EP300*, and *KMT2D* mutations tends to have inferior prognosis [10]. In a SAKK 38/07 prospective cohort, *CREBBP* mutation is an independent prognostic factor in DLBCL [11]. Elderly DLBCL patients are generally more frequently categorized into those high-risk groups [12, 13], providing clues for searching potential targeted therapeutic strategies.

Novel agents in combination with R-CHOP have been shown to improve clinical outcome of DLBCL. Ibrutinib plus R-CHOP prolongs PFS and OS in patients younger than 60 years [14]. Lenalidomide plus R-CHOP is effective in elderly patients with acceptable tolerability and mitigates the negative impact of non-GCB phenotype on patient prognosis [15]. Histone deacetylase inhibitors (HDACIs) are potent anti-lymphoma agents and synergistic activity between HDACIs and rituximab was observed [16]. Moreover, HDACIs sensitize B-lymphoma cells to chemotherapeutic agents [17] and clinical efficacy of HDACIs has also been investigated in patients with newly diagnosed DLBCL in combination with R-CHOP [18, 19].

Tucidinostat (formerly known as chidamide) is an oral benzamide class of HDACI that selectively inhibits Class I HDAC1, HDAC2, HDAC3, and Class IIb HDAC10, and has been applied to treat relapsed or refractory peripheral T-cell lymphoma as mono- or combinational therapy [20]. In the present study, a phase II study of chidamide plus R-CHOP (CR-CHOP) was conducted to evaluate the efficacy and safety in elderly patients with newly diagnosed DLBCL. Meanwhile, we performed whole genome sequencing (WGS) and targeted sequencing in patients with available tumor samples to explore relevance of molecular biomarkers in this prospective cohort.

## Results

### Patient characteristics

A total of 49 patients were included in the study between May 10, 2016 and May 2, 2018. Baseline characteristics are summarized in Table 1. Median age was 67 years (range 61–75), and 29 patients (59%) were male. Forty patients (82%) presented advanced Ann Arbor stage, and 41 patients (84%) showed elevated serum lactate dehydrogenase (LDH) level. Twenty-nine patients (59%) had multiple extranodal involvement, mainly as bone (35%), gastrointestinal (24%), and bone marrow (18%). Forty-two patients (86%) were of intermediate-high or high-risk of IPI at diagnosis. Twelve patients had BCL2/MYC double expression with exclusion of MYC and BCL2/BCL6 translocation.

**Table 1:** Baseline clinical and pathological characteristics

Characteristics	Enrolled patients (*n* = 49)
*Age (years)*	
Median	67 (61–75)
*Gender*	
Male	29 (59%)
Female	20 (41%)
*ECOG*	
0–1	38 (78%)
2	11 (22%)
*Ann Arbor stage*	
II	9 (18%)
III	15 (31%)
IV	25 (51%)
*LDH*	
Normal	8 (16%)
Elevated	41 (84%)
*Extranodal sites*	
0–1	20 (41%)
≥ 2	29 (59%)
*IPI*	
2	7 (14%)
3	17 (35%)
4	20 (41%)
5	5 (10%)
*Cell of origin*	
GCB	14 (29%)
non-GCB	35 (71%)
*Double expressor lymphoma*	
Yes	12 (25%)
No	37 (75%)

*ECOG* Eastern Cooperative Oncology Group, *LDH* lactate dehydrogenase, *IPI* International Prognostic Index

### Dose intensity

**Fig. 1:**
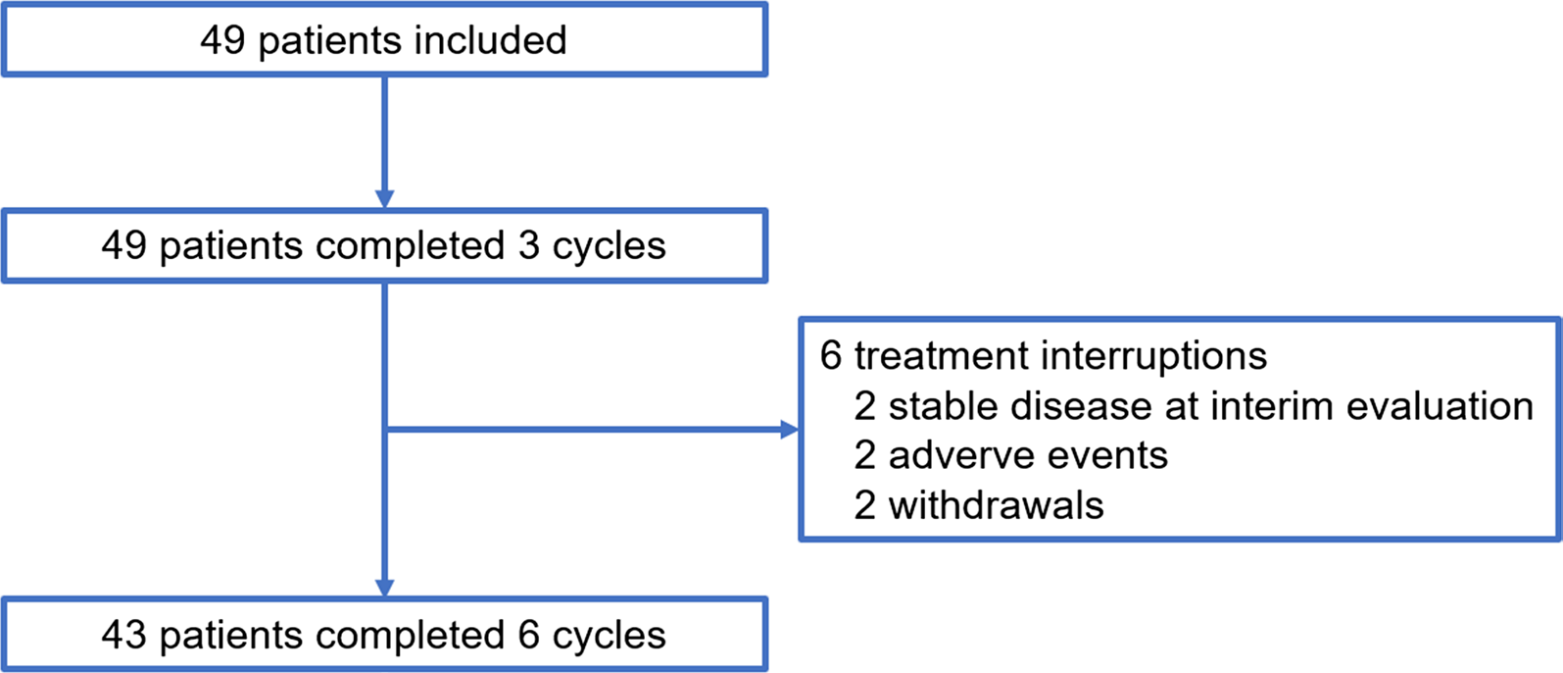
CONSORT diagram of the study

As shown in Fig. 1, 43 patients (88%) completed all six cycles of CR-CHOP. Two patients did not continue after the first three cycles because of stable disease. One patient withdrew consent after four cycles, and another three patients discontinued after five cycles: one withdrew consent, and two stopped due to adverse events (AEs), all of whom were in complete remission.

Of the 294 planned treatment cycles, 283 (96%) were given. The full dose was given in 266 (90%) of the 294 planned cycles. Tucidinostat postpones were applied in 8 cycles (3%), due to grade 4 neutropenia on day 11. Cyclophosphamide and doxorubicin dose reductions were applied in 15 cycles (5%), because of treatment delay due to grade 3 infections and grade 3–4 neutropenia. When analyzed by number of patients rather than number of cycles, four and eight patients had postpones of tucidinostat, and reduction of cyclophosphamide and doxorubicin, respectively.

### Response and survival

After completion of stage 1, 20 (87%, 95% CI 72–100) of 23 patients achieved complete response, justifying the initiation of stage 2 of Simon’s design. After completion of stage 2, 42 patients (86%, 95% CI 76–96) achieved complete response, and 4 patients (8%) achieved partial response. The four patients with partial response received additional involved-field radiotherapy to sites of residual uptake at final positron emission tomography-computed tomography (PET-CT), and one patient eventually achieved complete response. All three patients who did not respond after three cycles of treatment (including one patient with double hit lymphoma and one patient with triple-hit lymphoma) were salvaged with second-line chemotherapy and died from disease progression.

**Fig. 2:**
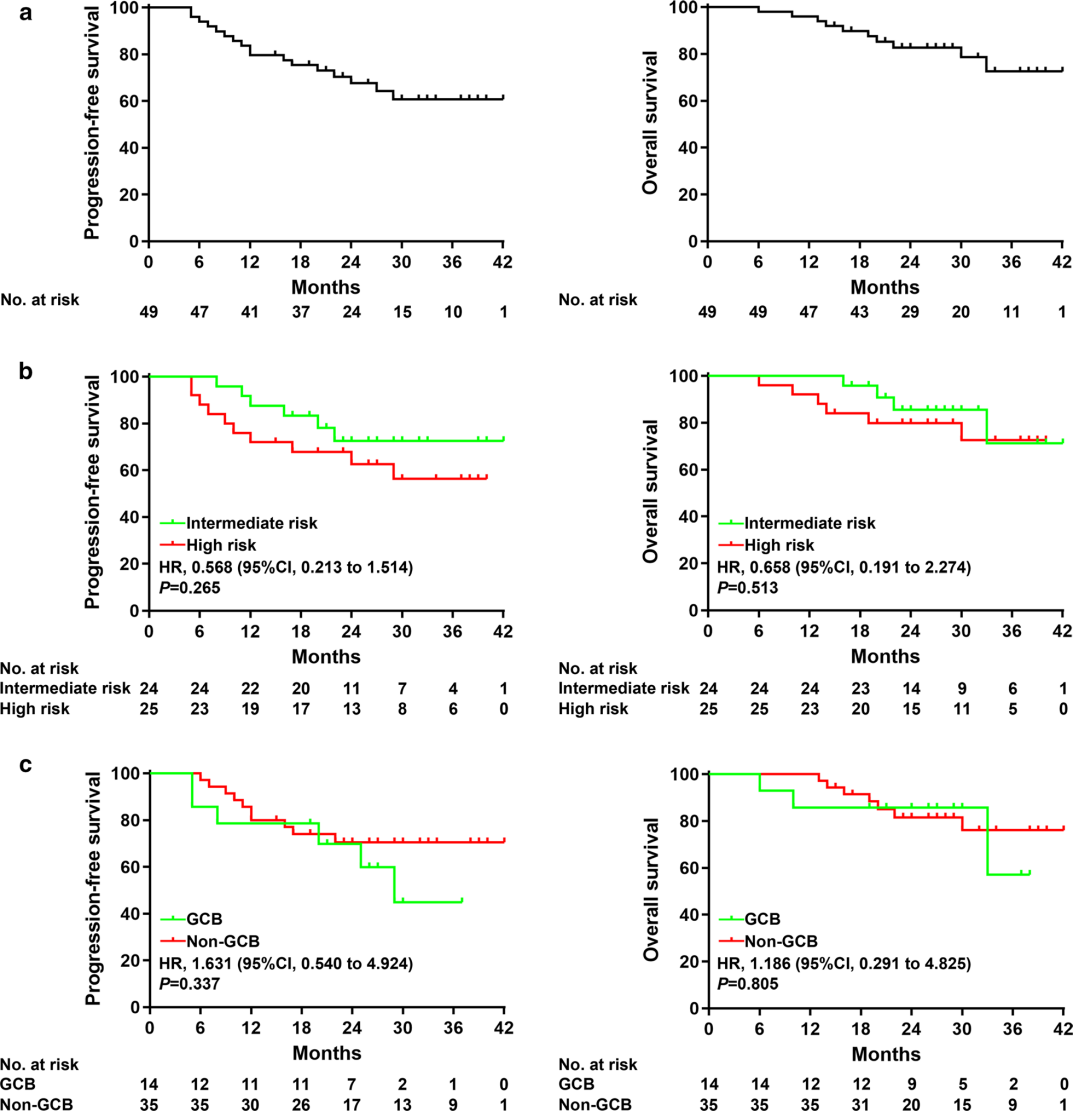
Outcomes of patients treated with CR-CHOP. **a** PFS and OS of all patients. **b** PFS and OS stratified by IPI. **c** PFS and OS stratified by cell of origin. CR-CHOP = tucidinostat (formerly known as chidamide), rituximab, cyclophosphamide, doxorubicin, vincristine, and prednisone. PFS = Progression-free survival. OS = Overall survival. IPI = International Prognostic Index

With a median follow-up of 30 months (range 6–42), the 2-year PFS and OS rates were 68% (95% CI 52–79) and 83% (95% CI 68–91), respectively (Fig. 2a). The 2-year PFS was 73% (95% CI 48–87) for patients with intermediate-risk (IPI 2–3) and 63% (95% CI 40–79) for those with high-risk (IPI 4–5) (HR 0.568, 95% CI 0.213–1.514; *P* = 0.265; Fig. 2b). The 2-year OS was 85% (95% CI 61–95) for patients with intermediate-risk and 80% (95% CI 58–91) for those with high-risk (HR 0.658, 95% CI 0.191–2.274; *P* = 0.513; Fig. 2b).

### Toxicity

Hematological and non-hematological AEs are summarized in Table 2. For hematological toxicities, grade 3 and 4 neutropenia were present in 31% and 53% of patients, respectively. Neutropenia was of short duration, with a median of 5 days (range, 3–7). Febrile neutropenia was reported in 15 cycles and was of grade 3 in maximum. Grade 3 and 4 thrombocytopenia were reported in 8% and 2% of patients, respectively, without bleeding complications. Grade 3 anemia was observed in 18% of patients. For non-hematological toxicities, grade 3 alanine aminotransferase (ALT) or aspartate aminotransferase (AST) elevation was observed in 8% of patients. No grade 4 non-hematologic toxicities were reported. Epstein–Barr virus DNA (EBV-DNA) was monitored routinely throughout the trial, and no positive event was reported.

**Table 2:** Hematological and non-hematological adverse events

Adverse events	Grade 1	Grade 2	Grade 3	Grade 4
*Number of cycles in which hematological adverse events were reported (n = 283)*				
Neutropenia	11 (4%)	28 (10%)	58 (20%)	113 (40%)
Thrombocytopenia	70 (25%)	48 (17%)	17 (6%)	10 (4%)
Anemia	85 (30%)	34 (12%)	11 (4%)	0
Febrile neutropenia	0	0	15 (5%)	0
*Number of patients with hematological adverse events (n = 49)*				
Neutropenia	2 (4%)	3 (6%)	15 (31%)	26 (53%)
Thrombocytopenia	12 (24%)	10 (20%)	4 (8%)	1 (2%)
Anemia	17 (35%)	12 (24%)	9 (18%)	0
Febrile neutropenia	0	0	6 (12%)	0
*Number of patients with non-hematological adverse events (n = 49)*				
Liver function abnormalities	5 (10%)	1 (2%)	4 (8%)	0
Infection	2 (4%)	6 (12%)	8 (16%)	0
Fatigue	2 (4%)	3 (6%)	0	0
Vomiting	3 (6%)	1 (2%)	0	0
Diarrhea	2 (4%)	1 (2%)	0	0
Hypoalbuminemia	5 (10%)	0	0	0
Hypokalemia	4 (8%)	0	0	0
Heart failure	0	1 (2%)	0	0
Atrial fibrillation	0	1 (2%)	0	0
Neurological	2 (4%)	0	0	0

No death occurred during the study as a result of toxicity related to treatment. Overall, nine patients died: eight for lymphoma progression, and one for severe pulmonary infection and heart failure in complete remission.

### Impact of phenotype on response to CR-CHOP

Twelve (86% [95% CI 65–100]) of the 14 patients with GCB achieved complete response, as did 30 (86% [95% CI 74–98]) of the 35 patients with non-GCB phenotype. The 2-year PFS was 70% (95% CI 38–88) in the GCB group and 71% (95% CI 52–83) in the non-GCB group (HR 1.631, 95% CI (0.540–4.924); *P* = 0.337; Fig. 2c). The 2-year OS was 86% (95% CI 54–96) in the GCB group and 81% (95% CI 63–91) in the non-GCB group (HR 1.186, 95% CI (0.291–4.825); *P* = 0.805; Fig. 2c). Outcomes for non-GCB patients tended to be improved upon CR-CHOP, as compared to historical control (2-year PFS and OS of 60% [95% CI 49–68] and 72% [95% CI 62–80], respectively).

**Fig. 3:**
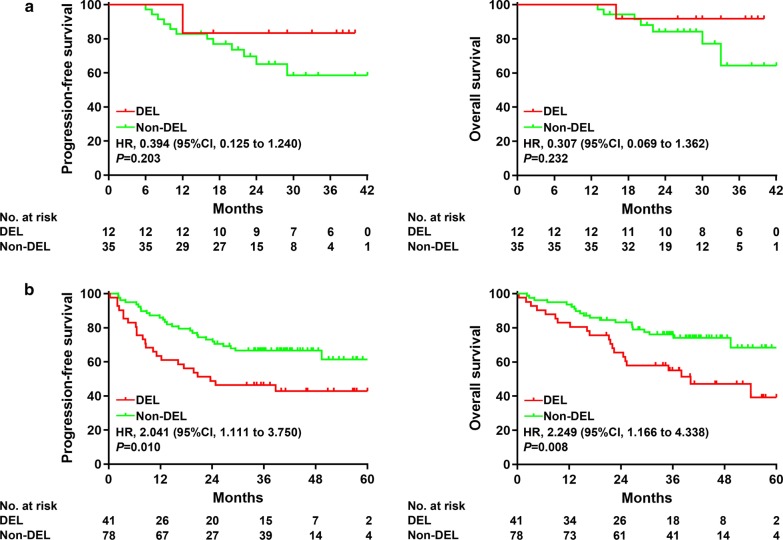
Outcomes of patients treated with CR-CHOP and historical control based on BCL2/MYC double expression. **a** PFS and OS of patients treated with CR-CHOP. **b** PFS and OS of patients from historical control (NCT01852435). CR-CHOP = tucidinostat (formerly known as chidamide), rituximab, cyclophosphamide, doxorubicin, vincristine, and prednisone. PFS = Progression-free survival. OS = Overall survival

All twelve patients (100%) with DEL phenotype achieved complete response, as did 30 (86% [95% CI 74–98]) of the 35 patients with non-DEL phenotype. The 2-year PFS and OS rates of patients with DEL phenotype were 83% (95% CI 48–96) and 92% (95% CI 54–79), which were comparable to those of non-DEL phenotype (PFS: HR 0.394, 95% CI 0.125–1.240, *P* = 0.203; OS: HR 0.307, 95% CI 0.069–1.362, *P* = 0.232; Fig. 3a). Instead, in historical control (NCT01852435) upon R-CHOP treatment, the 2-year PFS and OS rates of patients with DEL phenotype were 46% (95% CI 31–61) and 63% [95% CI 46–76], which were significantly lower than those of non-DEL phenotype (PFS: HR 2.041, 95% CI 1.111–3.750, *P*=0.010; OS: HR 2.249, 95% CI 1.166–4.338, *P* = 0.008; Fig. 3b).

### Impact of molecular alterations on response to CR-CHOP

**Fig. 4:**
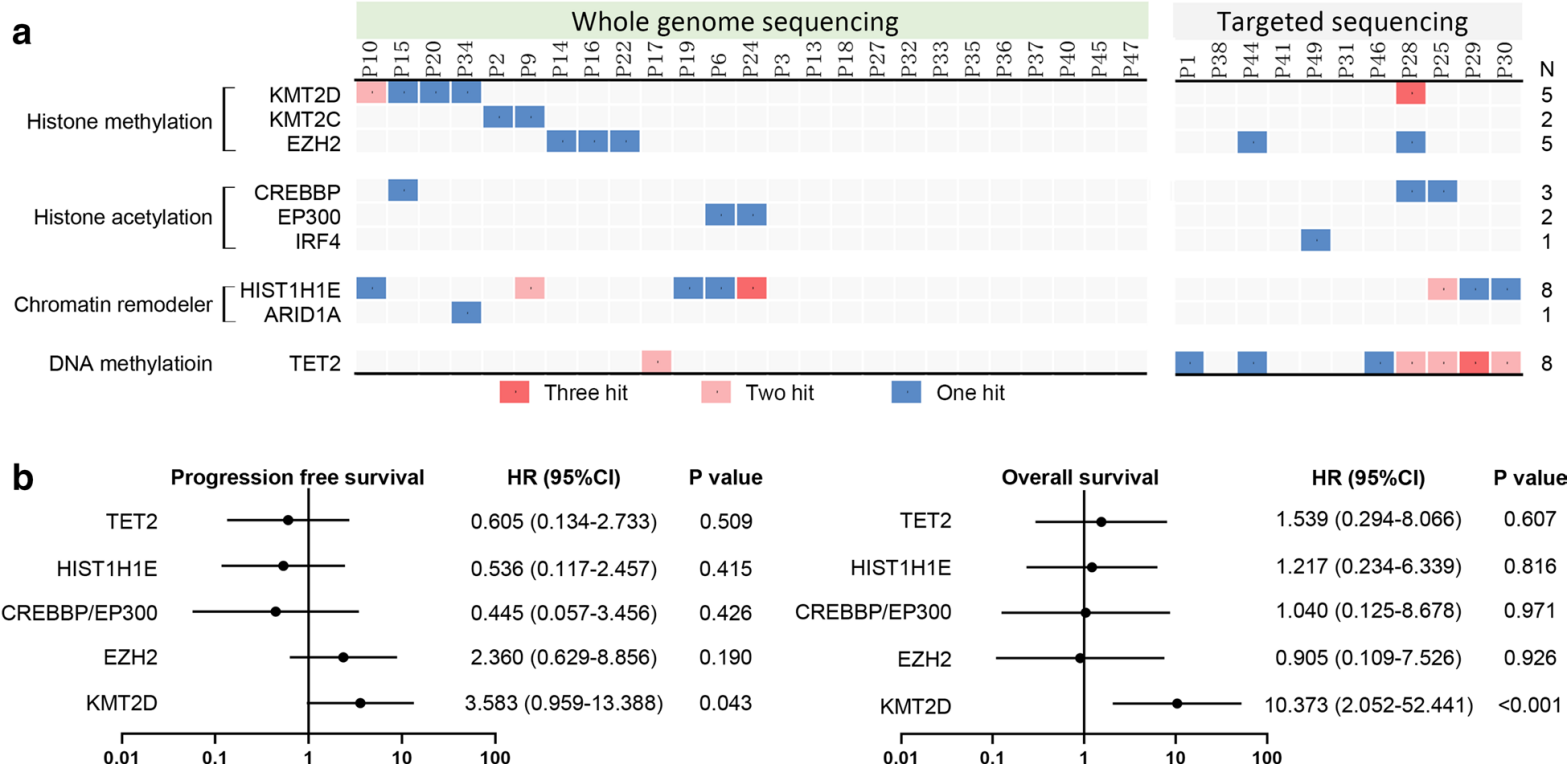
Correlation of genomic alterations with response to CR-CHOP. **a** Epigenetic gene mutations by whole genome sequencing and targeted sequencing in 36 patients. The number of patients with mutations was listed on the right. **b** Forest plot of univariate analysis on PFS and OS in patients with or without epigenetic gene mutations. PFS = Progression-free survival. OS = Overall survival

As shown in Fig. 4a, epigenetic gene mutations were identified in 21 of 36 (58%) patients, including genes related to histone methylation (*KMT2D* [5/36, 14%], *KMT2C* [2/36, 6%], and *EZH2* [5/36, 14%]), histone acetylation (*CREBBP* [3/36, 8%], *EP300* [2/36, 6%], and *IRF4* [1/36, 3%]), chromatin remodeler (*HIST1H1E* [8/36, 22%] and *ARID1A* [1/36, 3%]), and DNA methylation (*TET2* [8/36, 22%]). Overall, a total of 47 non-silent somatic mutations were identified, including 34 missense, 9 insertion or deletion, 4 nonsense, and a preference for C > T/A > G alterations analogous to the somatic single nucleotide variation (SNV) spectrum in other cancers.

In terms of the influence of epigenetic gene mutations on clinical outcome, mutations in *KMT2D*, but not in *CREBBP/EP300*, were associated with inferior PFS and OS (*P* = 0.043 and < 0.001, respectively, Fig. 4b). The 2-year PFS and OS rates were 40% (95% CI 5–75) and 40% (95% CI 5–75) in patients with *KMT2D* mutation, significantly shorter than those without mutation (68% [95% CI 47–82] and 89% [95% CI 70–96]). Although mutations in *CREBBP*/*EP300* were associated with inferior PFS and OS in patients of historical control (mutation rate 15%, PFS: HR 3.292, 95% CI 1.140–9.502, *P* = 0.028; OS: HR 4.628, 95% CI 1.379–15.530, *P* = 0.013), there was no significant difference of PFS and OS between patients with or without *CREBBP*/*EP300* mutation upon treatment with CR-CHOP (*P* = 0.426 and 0.971, respectively; Fig. 4b). The 2-year PFS and OS rates were 80% (95% CI 20–97) and 80% (95% CI 20–97) in patients with *CREBBP*/*EP300* mutation, 61% (95% CI 40–87) and 83% (95% CI 63–92) in patients without *CREBBP*/*EP300* mutation, respectively.

## Discussion

DLBCL in the elderly is associated with adverse clinical outcome and limited treatment options. In this phase II study, we showed that CR-CHOP was effective in first-line therapy of elderly patients with newly diagnosed DLBCL. After six cycles of CR-CHOP, the complete response rate was higher than those of previous reports in Western countries (71–76%) [2, 3] and in China (72%) [13], all of which contained low-risk IPI patients. In a recent phase I study of valproate plus R-CHOP in newly diagnosed DLBCL (26% of the patients had intermediate-high or high-risk IPI), improvement of clinical outcome has been achieved, with PFS of 84.7% and OS of 96.8% at 2 years [18]. The 2-year survival time was also encouraging with our study on CR-CHOP, considering that more than 80% of patients had intermediate-high or high-risk IPI. Of note, CR-CHOP was equally effective in high-risk IPI patients as intermediate-high IPI patients, and compared favorably to standard R-CHOP reported by LNH985 (2-year PFS and OS as approximately 50% and 60%) [2] and by our previous study (2-year PFS and OS as 41% and 63%, respectively) [13].

Another encouraging finding of our study was the clinical efficacy of CR-CHOP in the DEL phenotype of DLBCL. Though in historical control (NCT01852435), the outcome of DEL patients was significantly inferior to that of non-DEL patients upon R-CHOP treatment, no apparent difference was observed in patients treated with CR-CHOP. Improved outcomes were also reported for DEL in a phase I/II trial of vorinostat plus R-CHOP (SWOG S0806), as compared to those of R-CHOP (SWOG S0433) (2-year PFS 73% vs. 58%, 2-year OS 91% vs. 75%) [19]. These clinical findings are supported by experimental data showing that MYC acts as a biomarker of the anti-cancer action of HDACI romidepsin and entinostat in DLBCL [21, 22]. In other hematological malignancies, tucidinostat inhibits MYC and BCL2 in acute myeloid leukemia [23, 24], and tucidinostat also downregulates BCL2 in combination with doxorubicin in peripheral T-cell lymphoma [25]. Together, tucidinostat may improve the outcome of DLBCL patients through targeting MYC and BCL2. To further confirm the role of tucidinostat plus R-CHOP in DEL phenotype of DLBCL patients, a phase III, randomized, double-blind, placebo-controlled, multicenter study is currently ongoing in China (NCT04231448).

Histone modifying genes are critically involved in tumor progression of DLBCL [26]. Inactivation of *CREBBP* and *EP300* abrogates germinal center B cell formation and contributes to lymphomagenesis, as revealed by conditional germinal center-directed deletion mouse models targeting Crebbp or Ep300 [27]. Clinically, *CREBBP* mutation is an independent prognostic factor in DLBCL in a prospective SAKK 38/07 trial [11]. Disruption of *KMT2D* also perturbs germinal center B cell development [28, 29] and *KMT2D* mutations are the most frequent relapse-specific events in DLBCL [30]. In consistence with experimental data showing that HDACIs can rescue deficits in histone acetylation induced by *CREBBP/EP300* mutations in B-cell lymphoma [26], our results provided clinical evidence that tucidinostat may mitigate the negative prognostic impact of *CREBBP*/*EP300* mutations on DLBCL. However, mutations in *KMT2D* remained to be an adverse prognostic factor in DLBCL treated with CR-CHOP, which could be alternatively targeted by hypomethylating agents [31].

Safety profile is a major issue for the addition of novel agent to an existing combination, including HDACIs. Vorinostat plus R-CHOP is associated with high rates of AEs in patients with newly diagnosed advanced stage DLBCL (SWOG S0806) [19]. Also, unexpected auditory AEs occur in valproate plus R-CHOP [18]. Here, we recorded no grade 4 non-hematological AEs and no deaths due to toxicity. Grade 3–4 neutropenia was recorded in almost 20%–40% of cycles despite the use of granulocyte-colony stimulating factor (G-CSF), but it was of short duration and did not translate into an excess of infections. Most AEs in our trial were of mild-to-moderate intensity, and similar to those recorded with standard R-CHOP in elderly patients with DLBCL. No new clinically significant toxicity was noted with the addition of tucidinostat to R-CHOP. EBV reactivation is of concern in the administration of HDACI [32] and was not observed in this study.

With regard to dose intensity and the feasibility of the CR-CHOP regimen, the analysis of drug delivery showed that patients received full dose of tucidinostat and at least 90% of the doses of R-CHOP drugs in more than 90% of cycles. This frequency is acceptable when comparing with other studies of standard R-CHOP, in which the percentage of the patients who received more than 90% of the dose intensity of the drugs varied from 64 to 90% [14, 15]. Therefore, the addition of tucidinostat does not impair full delivery of R-CHOP, provided that G-CSF is used.

## Conclusion

CR-CHOP was well tolerated and showed promising clinical activity in DLBCL. Our encouraging data warrant validation in a future phase III randomized trial to elucidate the relevance of DEL phenotype and molecular biomarkers on response to R-CHOP in combination with tucidinostat in DLBCL.

## Methods

### Eligibility criteria

Patients were eligible if they were 61–75 years; had newly diagnosed, histologically confirmed CD20-positive DLBCL, an Eastern Cooperative Oncology Group (ECOG) performance status of 0–2, IPI risk of intermediate or high (IPI ≥ 2), and a life expectancy of more than 6 months. Patients were excluded if they had previous chemotherapy or stem cell transplantation; history of malignancy (other than skin cancers or carcinomas in situ of the cervix); uncontrollable cardio-cerebral vascular, coagulation, autoimmune, infectious disease; primary central nervous system (CNS) lymphoma; left ventricular ejection fraction ≤ 50% [4, 33]. They were also eliminated from the study if, at enrollment, their neutrophil count < 1.5 × 10^9^/L, platelets < 75 × 10^9^/L, ALT or AST > 2 × upper limit of normal (ULN), AKP or bilirubin > 1.5 × ULN, or creatinine > 1.5 × ULN (unless they were caused by the lymphoma). They were not enrolled if they were not able to comply with the protocol for mental or other unknown reasons; pregnancy or lactation; or positive for hepatitis B virus (HBV-DNA) and human immunodeficiency virus.

Pathological diagnosis was performed according to the 2016 World Health Organization classification [1]. Cell of origin profile was determined by Hans algorithm, with 30% cutoff value of CD10, BCL6, and MUM-1 [6]. As for DEL phenotype, cutoff value of BCL2 and MYC was 50% and 40%, respectively [1]. Fluorescence in situ hybridization of BCL2, BCL6, and MYC rearrangements was performed for each patient.

### Study design and procedures

This was an investigator-initiated, open-label, single-arm, phase II study. The dose and administration schedule of CR-CHOP were as follows: rituximab 375 mg/m^2^ given intravenously on day 0, cyclophosphamide 750 mg/m^2^, doxorubicin 50 mg/m^2^, and vincristine 1.4 mg/m^2^ (maximum 2.0 mg) intravenously on day 1, prednisone 60 mg/m^2^ (maximum 100 mg) orally on days 1–5, and tucidinostat 20 mg orally on days 1, 4, 8, and 11, according to the maximum tolerated dose in combination with CHOP and etoposide in peripheral T-cell lymphoma (ClinicalTrials.gov Identifier: NCT02987244) [34]. The regimen was repeated every 21 days with a total of six cycles.

Tucidinostat should be postponed on the occurrence of grade ≥ 3 hematological or non-hematological toxicities. There was no plan for dose reduction of tucidinostat. The administration was resumed when the AEs were to grade 1 or pre-treatment levels. G-CSF prophylaxis (recombinant human pegylated G-CSF of 100 ug/kg) was given from the second cycle of chemotherapy if grade ≥ 3 neutropenia was present in the first cycle. Lamivudine was administered in occult carriers of HBV to prevent HBV reactivation. Prophylaxis for CNS relapse was given to patients with involvement of bone marrow, nasal or paranasal sinuses, orbit, breast, or testis. Tumor lysis prophylaxis and radiation therapy were performed for patients with bulky disease, or with residual disease at the end of treatment, at the discretion of physicians.

Baseline evaluations were performed within 28 days prior to therapy, including physical examination, complete blood cell count, serum biochemistry with LDH, coagulation function, HBV markers and DNA, EBV-DNA, electrocardiogram, echocardiography, bone marrow aspiration and trephine biopsy, and PET-CT. The patients were staged according to Ann Arbor staging system, and IPI [35] was calculated. Treatment response was assessed according to the standardized response criteria established by Cheson et al. [36]. Interim efficacy was evaluated by PET-CT after three cycles. Patients who had achieved a complete or partial response received another three cycles. Patients who did not achieve a complete or partial response stopped receiving CR-CHOP at this point. Final evaluation was performed by PET-CT one month after the end of the last cycle of treatment. CT of the neck, thorax, abdomen, and pelvis was repeated every 3 months thereafter to monitor disease progression until 1 year, then every 6 months until 2 years, and every year thereafter.

### Study end points and assessments

The primary endpoint was complete response rate assessed by PET-CT. Secondary endpoints were PFS, OS, overall response rate, and AEs. PFS was measured from diagnosis to date of progression, relapse, or death from any cause. Patients with a partial response who were given an additional treatment (i.e., radiation therapy) without apparent disease progression were not considered as an event for PFS analysis. OS was measured from diagnosis to death of any cause or date of last follow-up. AEs were categorized and graded according to the National Cancer Institute Common Terminology Criteria for Adverse Events (CTCAE, version 4.0).

### Historical control cohort

Elderly patients from 20 centers of the Multicenter Hematology/Oncology Programs Evaluation System (M-HOPES) in China with newly diagnosed DLBCL, treated with regular R-CHOP50 (doxorubicin 50 mg/m^2^) or regular R-CEOP70 (rituximab, cyclophosphamide, epirubicin 70 mg/m^2^, vincristine, and prednisone) between May 15, 2013 and March 16, 2016 in our previous multicenter, phase III, randomized, controlled trial (NCT01852435) [13], and who met the same inclusion/exclusion criteria as those treated with CR-CHOP were referred as the historical control cohort and analyzed for outcome on the basis of DLBCL subtypes.

### DNA sequencing

WGS was performed on frozen tumor samples of 25 patients with genomic DNA extracted using Wizard® Genomic DNA Purification Kit (Promega, Wisconsin-Madison, USA). The depth of samples measured with WGS was 50–200× , with 83–99% of the target sequence being covered sufficiently deep for variant calling (≥ 10 × coverage). A total of 9 genes reported to be hypermutation (> 5%) or related to lymphoma pathogenesis in DLBCL were selected. SNVs and indels were called by Genome Analysis Toolkit (GATK, v3.7.0) Haplotype Caller and GATK Unified Genotyper, and mapped to the genome location using the UCSC Genome Browser (https://genome.ucsc.edu/). The reference genome was the Refseq database (Human Reference Genome version hg19). All the somatic functional mutations, including non-synonymous SNVs, frameshift or in-frame indels, stopgain or stoploss, were obtained. Visual inspection was used to exclude potential false positive results. Homemade pipeline was used to filter SNVs and indels detected by the above software, excluding: (1) mutations reported with low confidence; (2) germline mutations detected from control samples; (3) population-related variants reported in 1000 Genomes (dbSNP 138) as common SNPs and not included in COSMIC (the Catalogue of Somatic Mutations in Cancer) version v77.

Targeted sequencing was performed on formalin-fixed paraffin-embedded tumor samples of 11 patients with genomic DNA extracted using GeneRead DNA formalin-fixed paraffin-embedded Tissue Kit (Qiagen, Hilden, Germany). The depth of samples measured with targeted sequencing was 1000–2000× , with 85–98% of the target sequence being covered sufficiently deep for variant calling (≥ 10× coverage).

### Statistical analysis

This phase II study was designed according to Simon’s two-stage minimax design [37]. Our objective was to show a 15% improvement in frequency of complete response with the new regimen relative to an expected frequency of 70% with R-CHOP alone [38], with an α of 0.05, 80% power. A total of 49 patients were required to obtain the hypothesis, 23 of whom were to be enrolled during stage 1 and 26 during stage 2. The study would be stopped early if fewer than 16 (70%) of the patients achieved complete response in stage 1. Similarly, if 39 (80%) or fewer patients achieved complete response by trial completion, the hypothesis would also be rejected.

Efficacy and safety analyses were by intention to treat. Statistical analyses were performed by Statistical Package for the Social Science (SPSS) 23.0 software (SPSS Inc., Chicago, IL, USA). Survival estimates were calculated by Kaplan–Meier method, and survival curves were compared by log-rank test. A two-sided P value of < 0.05 was considered statistically significant.

## Data Availability

The datasets used and/or analyzed during the current study are available in Mendeley Data through the 10.17632/nmxgxsvbk5.1 and National Omics Data Encyclopedia (NODE, https://www.biosino.org/node/) under Accession Number OEP001040.

## Abbreviations

DLBCL: Diffuse large B-cell lymphoma
IPI: International Prognostic Index
PFS: Progression-free survival
OS: Overall survival
R-CHOP: Rituximab, cyclophosphamide, doxorubicin, vincristine, and prednisone
GCB: Germinal center B-cell
ABC: Activated B-cell
DEL: Double-expressor lymphoma
HDACI: Histone deacetylase inhibitor
CR-CHOP: Tucidinostat (formerly known as Chidamide), rituximab, cyclophosphamide, doxorubicin, vincristine, and prednisone
WGS: Whole genome sequencing
LDH: Lactate dehydrogenase
AE: Adverse event
PET-CT: Positron emission tomography-computed tomography
ALT: Alanine aminotransferase
AST: Aspartate aminotransferase
EBV-DNA: Epstein–Barr virus DNA
SNV: Single nucleotide variation
G-CSF: Granulocyte-colony stimulating factor
ECOG: Eastern Cooperative Oncology Group
CNS: Central nervous system
ULN: Upper limit of normal
HBV-DNA: Hepatitis B virus DNA
CTCAE: Common Terminology Criteria for Adverse Events
